# Fractal Patterns of Neural Activity Exist within the Suprachiasmatic Nucleus and Require Extrinsic Network Interactions

**DOI:** 10.1371/journal.pone.0048927

**Published:** 2012-11-20

**Authors:** Kun Hu, Johanna H. Meijer, Steven A. Shea, Henk Tjebbe vanderLeest, Benjamin Pittman-Polletta, Thijs Houben, Floor van Oosterhout, Tom Deboer, Frank A. J. L. Scheer

**Affiliations:** 1 Medical Biodynamics Program, Division of Sleep Medicine, Brigham and Women's Hospital, Boston, Massachusetts, United States of America; 2 Medical Chronobiology Program, Division of Sleep Medicine, Brigham and Women's Hospital, Boston, Massachusetts, United States of America; 3 Division of Sleep Medicine, Harvard Medical School, Boston, Massachusetts, United States of America; 4 Department of Molecular Cell Biology, Laboratory for Neurophysiology, Leiden University Medical Centre, Leiden, The Netherlands; 5 Center for Research on Occupational and Environmental Toxicology, Oregon Health & Science University, Portland, Oregon, United States of America; Wake Forest University, United States of America

## Abstract

The mammalian central circadian pacemaker (the suprachiasmatic nucleus, SCN) contains thousands of neurons that are coupled through a complex network of interactions. In addition to the established role of the SCN in generating rhythms of ∼24 hours in many physiological functions, the SCN was recently shown to be necessary for normal self-similar/fractal organization of motor activity and heart rate over a wide range of time scales—from minutes to 24 hours. To test whether the neural network within the SCN is sufficient to generate such fractal patterns, we studied multi-unit neural activity of *in vivo* and *in vitro* SCNs in rodents. *In vivo* SCN-neural activity exhibited fractal patterns that are virtually identical in mice and rats and are similar to those in motor activity at time scales from minutes up to 10 hours. In addition, these patterns remained unchanged when the main afferent signal to the SCN, namely light, was removed. However, the fractal patterns of SCN-neural activity are not autonomous within the SCN as these patterns completely broke down in the isolated *in vitro* SCN despite persistence of circadian rhythmicity. Thus, SCN-neural activity is fractal in the intact organism and these fractal patterns require network interactions between the SCN and extra-SCN nodes. Such a fractal control network could underlie the fractal regulation observed in many physiological functions that involve the SCN, including motor control and heart rate regulation.

## Introduction

In mammals, many physiological and behavioral variables, including heart rate and motor activity, exhibit temporal structures that are similar across widely different time scales, i.e. “fractal” or “scale-invariant” patterns [1], [2]. Fractal patterns of heart rate and motor activity levels are intrinsic system characteristics that are independent of environmental and behavioral stimuli [2], [3]. These fractal controls appear to impart health advantages, including system integrity and adaptability [4]. For instance, fractal cardiac and activity controls are reduced with aging and under pathological conditions [1], [5], and the degree of reduction in fractal cardiac control can be predictive of survival [6].

The physiological mechanisms responsible for such fractal regulation remain unknown. However, we recently discovered in rodents that the master clock of the circadian system (suprachiasmatic nucleus; SCN) [7] is essential for the overall expression of normal fractal patterns in motor activity fluctuations over a wide range of time scales from minutes to ∼24 hours [8]. These fluctuation patterns cannot be generated by a simple superposition of independent oscillations at different time scales [9], and require feedback interactions between control nodes that affect a physiological system at multiple time scales [2], [8]. The SCN is comprised of a network of thousands of heterogeneous neurons (∼20,000 in rodents and ∼80,000 in humans) [10]–[12], raising the possibility that the SCN itself has sufficient complexity to generate these fractal patterns. Alternatively, the SCN may be only part of a larger network that also includes non-SCN control nodes in order to generate such fractal patterns. Thus, using long-term *in vivo* multi-unit neural activity (MUA) recordings of the SCN in freely moving rodents, we tested the hypothesis that SCN neural activity exhibits a similar fractal pattern as observed in motor activity. Second, by examining MUA in rodents kept in constant darkness, we tested whether or not the main afferent input to the SCN, namely light, is required for the generation of this fractal pattern *in vivo*. Third, by recording *in vitro* MUA in an SCN slice preparation in which synchronized circadian rhythmicity in neural activity persists, we tested whether the fractal pattern is a network property within the SCN itself, or whether this pattern requires extrinsic network interactions between the SCN and control nodes outside the SCN. Fourth, by analyzing MUA recordings from both mice and rats, we tested whether the SCN activity possesses similar fluctuation patterns in two rodent species. Finally, we tested a secondary hypothesis that motor activity fluctuations in mice also display similar fractal patterns as we previously observed in motor activity fluctuations of humans and rats [2], [8]. Our previous findings of the relevance of the SCN to fractal regulation were mainly based on studies of humans and rats [2], [5], [8]. Testing the last two hypotheses will allow us to determine whether genetic mouse models can be used in future studies to better understand the neural circuitry of fractal regulation.

## Methods

### Ethics Statement

All data were collected in Laboratory for Neurophysiology, Leiden University Medical Centre, Leiden, the Netherlands. The animal handling procedures and research protocols were approved by the Animal Experiments Ethical Committee of the Leiden University Medical Center with DEC nr 4085.

### Animals

To test our hypotheses, we analyzed MUA of the SCNs of 15 adult C57BL6 mice (Harlan, Horst, the Netherlands) and 16 adult Wistar rats (Harlan, Horst, the Netherlands). For all comparisons, data were grouped within each species and compared between species.

### Protocol

MUA recordings *in vivo* were continuously collected in 8 mice and 10 rats during two protocols: (i) light-dark (LD; 12 hours∶12 hours) cycles (6 mice and 5 rats; duration range of individual recordings: 66–168 hours; total recording duration: ∼984 hours), and (ii) constant dark condition (DD) (7 mice and 6 rats; duration range: 42–259 hours; total recording duration: ∼1,713 hours). The light intensity was ∼150 lux during the light phases, and 0 lux during the dark phases or constant darkness. Not all animals went through both LD and DD protocols. During the LD and DD protocols, the animals had free access to water and food [Food type: 801203RM3(P)PL. IRR, from Special Diets Service, Essex, England].

### *In vivo* MUA

**Figure 1:**
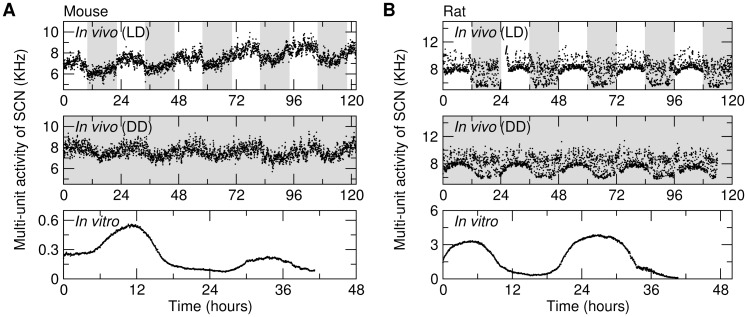
Multi-unit activity recordings of the SCNs from mice and rats. Representative recordings *in vivo* from (A) a mouse and (B) a rat during LD (Top panels) and DD (Middle panels), and representative recordings *in vitro* of a mouse and a rat (Bottom Panels). Gray bar indicates dark condition for *in vivo* recordings.

*In vivo* MUA (Figure 1) was recorded from animals using two tripolar stainless steel electrodes (125 µm, Plastics One, Roanoke, VA) that were implanted in the brain under a 5° angle in the coronal plane using a stereotactic device and bilaterally aimed at the SCN [13]. A third electrode was cut shorter with the insulation at the distal end removed, and was placed in the white matter to electrically ground the animals. Following the surgery, animals were allowed to recover for at least one week. At the onset of the experimental protocol, the animals were connected to the recording system via a flexible cable that was attached to a counterbalanced swivel system, thereby permitting substantial freedom to move. The signals from the recording electrodes were amplified and filtered [14]. The number of action potentials crossing a preset threshold were counted by a computer in 10 second bins (sampling frequency = 0.1 Hz) and stored for off-line analysis.

### Motor activity

To test our secondary hypothesis that motor activity fluctuations in mice display fractal patterns similar to those we have observed in the motor activity fluctuations of rats and humans [2], [8], we also analyzed a total of ∼1,539 hours of motor activity data from 5 mice (∼1,083 hours from 3 mice during the DD and ∼456 hours from 5 mice during the LD) that were collected from a passive infrared sensor located in the ceiling of the recording cage (see Figure S1). The sensor detected movements of the mouse across the surface of the cage (40×40 cm) and data were integrated over 10-second epochs. Note that MUA recordings showing behaviorally induced suppressions of SCN neural activity (estimated from motor activity) were excluded in the study [15].

### *In vitro* MUA

MUA from SCN brain slices was recorded in 7 mice and 6 rats as described previously [16]. Briefly, ∼500-µm coronal slices containing the SCN were prepared, submerged in a laminar flow chamber and perfused continuously with artificial cerebrospinal fluid of 35.5°C. All SCN slices contained ≥40% (Mean ± SE: 67%±7%) of the SCN in the rostro-caudal plane (Table S1) [17]. For each slice, extracellular electrical activity was recorded by two stationary electrodes (75 µm, 90% platinum, 10% iridium), amplified 10 k times, and band-pass filtered (300 Hz low, 3 kHz high). The action potentials crossing a preset threshold well above noise (∼5 µV) were counted electronically in 10-second bins (sampling frequency = 0.1 Hz) for ≥36 hours. Time of occurrence and the amplitudes of action potentials, as well as action potential waveforms were digitized by a Power1401 (CED, Cambridge, U.K.) and stored for off-line analysis. *In vitro* recordings started ∼1 hour (±5 minutes) after harvesting the SCN (Figure 1). Total ∼527 hours of data were collected (∼256 hours from 6 rats and ∼271 hours from 7 mice).

### Activity of single units and subpopulations of the *in vitro* SCN

To study small populations of SCN neurons *in vitro*, we performed an offline subpopulation analysis of the action potentials of 6 recordings from 4 *in vitro* experiments (2-channel recordings for two experiments and 1-channel for the other two experiments). In this analysis we used a higher sampling resolution (sampling frequency = 1.0 Hz). This analysis allows for selection of the size of the population of SCN neurons through use of varied voltage thresholds [18]. We selected voltage thresholds such that the average activity in the 30-minute window centered at the MUA peak was close to a targeted level (2.5, 5.0, 7.5, and 10.0 Hz) with a deviation <0.7%. For each of the three voltage thresholds, we obtained ∼182 hours of subpopulation data from the four experiments using MATLAB 6 (MathWorks, Inc., Natick, Massachusetts, USA). Additionally, we isolated the activity profiles of two single units from two subpopulation recordings using clustering techniques (total ∼38 hours) [18], [19].

### Fractal analysis

To assess the scale-invariant/fractal structure in the fluctuations of SCN neural activity, we used detrended fluctuation analysis (DFA) [1], [2], [5]. The DFA was designed to identify fractal correlations in signals with embedded nonstationarities or trends (i.e., signals with statistical properties such as mean and standard deviation that vary with time). The method quantifies the detrended fluctuation amplitude of a signal at different time scales. For each chosen time scale n, the DFA method involves the following steps: (i) integrating the time series; (ii) dividing the integrated time series into non-overlapped windows of equal size ‘n’ (the chosen time scale); (iii) in each window, fitting the integrated time series with a second order polynomial function, which defines ‘local’ trends (second order polynomial functions were used to better remove trends in original data [2], [5]); (iv) detrending the integrated time series by subtracting the local trends; and (v) calculating the root mean square of the residuals in all windows to obtain the average fluctuation amplitude. The above procedure is repeated for different time scales *n* to obtain the detrended fluctuation function *F(n)*.

**Figure 2:**
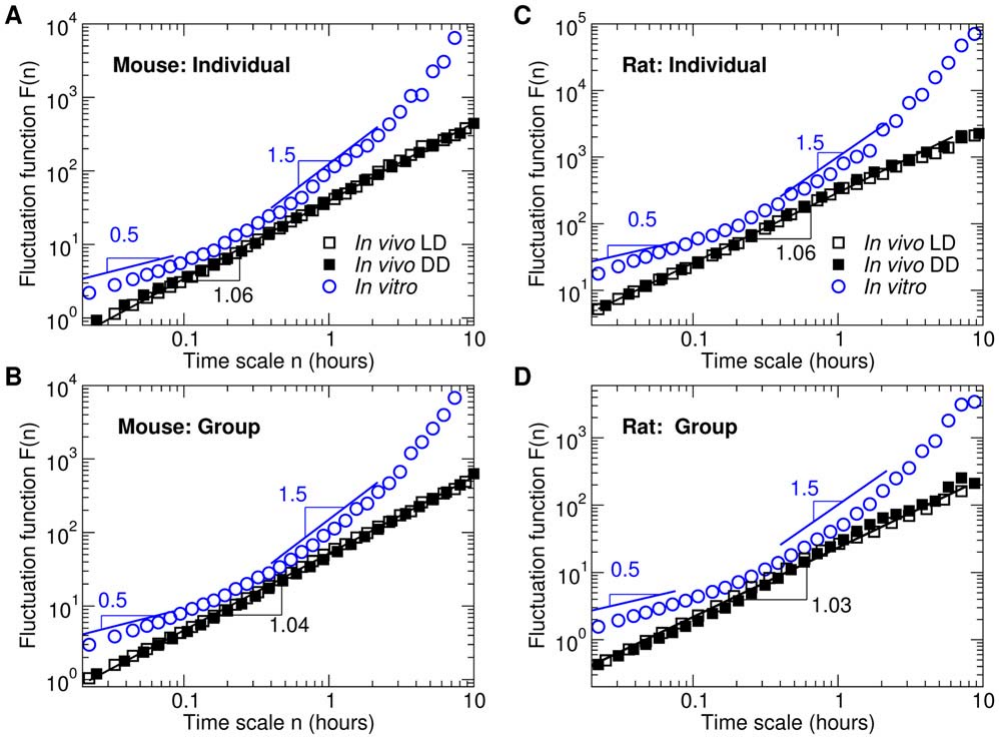
The fractal patterns of multi-unit neural activity fluctuations *in vivo* and the non-fractal patterns of multi-unit activity fluctuations *in vitro*. (**A**) Results of two representative individual mice: one for *in vivo* recordings during LD and DD and one for the *in vitro* recording. Corresponding raw data are shown in Figure 1A. (**B**) The group averages of mice. (**C**) Results of two representative individual rats: one for *in vivo* recordings during LD and DD and one for the *in vitro* recording. Corresponding raw data are shown in Figure 1B. (**D**) The group averages of rats. Data were shown in log-log plots and were vertically shifted for a better visualization of differences between the *in vivo* and *in vitro* recordings. At time scales from ∼1 minute up to ∼10 hours, the function *in vivo* shows a power-law form (straight line in the log-log plot) with the scaling exponent α≈1.0, indicating strong fractal correlations in raw data. For the group averages, the data of each subject were normalized to account for individual differences in the standard deviation of multi-unit activity. The fractal pattern of the *in vivo* recordings is virtually identical during LD and DD and is consistent for both mice and rats. In contrast, the fluctuation function of multi-unit neural activity *in vitro* did not have a power-law form, indicating complete loss of the scale-invariant/fractal correlations. The non-fractal pattern of the *in vitro* activity is virtually identical for mice and rats, showing a local slope close to 0.5 at time scales of 1–6 minutes and >1.5 at time scales of 2–5 hours.

A fractal structure in fluctuations is indicated by a power-law functional form (Figure 2), *F(n)*∼*n* ^α^, which is a straight line on a log-log plot of *F(n)* versus time scale *n*. The parameter α, called the scaling exponent, quantifies the correlation properties in the signal: if α = 0.5, there is no correlation in the fluctuations (random noise); if α>0.5, there are positive correlations, where large activity values are more likely to be followed by large activity values (and *vice versa* for small activity values). When α is very large (close to and greater than 1.5), the signal is characterized by predictable fluctuation patterns resulting in strong correlations (as seen in Brownian motion with α = 1.5 ) [4]. The most interesting, complex behavior is associated with an α of ∼1.0 which, as observed in non-equilibrium physical systems and most healthy physiological systems, indicates a fine balance between uncorrelated randomness and excessive regularity [4]. Under pathological conditions where this balance is perturbed, physiological fluctuations can become either too random (e.g., α of heartbeat fluctuations approaches 0.5 for atrial fibrillation) or too predictable (e.g., α of heartbeat fluctuations≈1.5 for congestive heart failure) [4].

In contrast, a non-power-law form of the fluctuation function F(n) (i.e., not a straight line in a log-log plot) reveals a lack of fractal correlations in the fluctuations, indicating either influences on the variable from only one source operating at one specific time scale, or simple additive influences from a number of independent control nodes without interactions.

For a reliable estimation of F(n) at a specific time scale n, the DFA requires at least 4 (ideally 10) non-overlapping segments of size n without missing data [20]. In 7 *in vivo* MUA recordings collected from rats during the DD, there were missing data points every few minutes. In order to assess fractal patterns at time scales up to 5 hours, we down-sampled these recordings using epoch length = 600 seconds (see Text S1). For each down-sampled signal, we had at least six 5-hour non-overlapping segments without gaps. The possible combined effects of the missing data and the compensatory down-sampling procedure were estimated by simulations and the scaling exponents were adjusted accordingly for these 7 recordings (see Text S1).

### Statistical analysis

The primary variable is the scaling exponent α that characterizes the fractal correlations of MUA. The secondary variables include deviations of the detrended fluctuation function from power-law fit, circadian amplitude, and MUA variations at smaller time scales (≤12 hours). To assess the differences between the LD and DD conditions, mixed model ANOVAs were performed to account for individual difference in mean level (‘intercepts’). Similar mixed model ANOVAs were used to test the difference between the light and dark portions of the LD protocol. ANOVAs were applied to determine the differences between *in vivo* MUA and *in vitro* MUA and between mice and rats. All statistical procedures were performed using JMP version 9.0 (SAS Institute Inc, North Carolina). Statistical significance was set at p<0.05.

## Results

### *In vivo* MUA exhibits fractal patterns during light-dark cycles

To test the existence of fractal patterns of *in vivo* MUA recordings, we used detrended fluctuation analysis (see Methods). By separately analyzing data collected from both mice and rats during light-dark cycles (LD: 12 h∶12 h; Figure 1), we found that the fluctuation function *F(n)* of the *in vivo* MUA possessed a power-law form (a straight line in the log-log plot: *F(n)∼n* ^α^) at time scales from ∼1 minute up to 10 hours, i.e., spanning a range of more than two orders of magnitude (Figure 2). The power-law form indicates a fractal temporal structure and the scaling exponent α of ∼1.0 (mean±SE, mice LD: 1.04±0.03; rats LD: 1.08±0.03) indicates strong fractal correlations in MUA fluctuations. The long-range fractal correlations in the *in vivo* MUA fluctuations were similar between mice and rats (p>0.5). In addition, the fractal patterns of MUA were similar to those observed in motor activity of humans and rats [2], [8] as well as to those observed in motor activity data collected from a subgroup of mice in this study (see Text S2; Figure S1).

### Fractal patterns of *in vivo* MUA are independent of afferent light input to the SCN

Light, through the activation of retinal ganglion cells, is the main time cue of the circadian system, which can affect SCN activity acutely and also reset its circadian phase [13]. Although the illumination level was constant in the light portion of the LD protocol, light input to the retina is unlikely to be constant and could display complex patterns, for instance due to diminished light input during the frequent sleep episodes of variable duration that typically occur in rodents [21]. Thus, to test whether or not the fractal pattern of *in vivo* MUA was caused by the light-induced variation in afferent input to the SCN, we analyzed ∼2,253 hours of *in vivo* MUA recordings collected from mice and rats during constant darkness (DD) (Figure 1). *In vivo* MUA during DD also displayed a power-law fluctuation function F(n) with no significant change in scaling exponent as compared to that observed during LD (mice DD: 1.04±0.03; rats DD: 1.03±0.07) (LD vs DD: p>0.7; Figure 2). Furthermore, separate analysis of the light and the dark portions of the LD protocol revealed similar power-law forms during the light and dark portions (mice: L, 1.02±0.03; D, 1.04±0.03; rats: L, 1.05±0.04; D, 1.09±0.05; L vs D: p = 0.19 [with no interaction effect regarding species], see Figure S2). Thus, the fractal fluctuations of *in vivo* MUA cannot be attributed to light-elicited variability in the afferent input to the SCN.

### Fractal patterns in MUA are completely abolished in the *in vitro* SCN preparation despite persistence of circadian rhythmicity in average MUA

To test whether the observed fractal correlations in MUA require network interactions between the SCN and control nodes outside the SCN, we analyzed ∼527 hours of *in vitro* MUA recordings collected from mice and rats (Figure 1). The *in vitro* SCN slice preparation was viable as indicated by persistent circadian rhythmicity of MUA (see Text S3). However, the fluctuation function, *F*(*n*), of *in vitro* MUA was dramatically different from that of *in vivo* MUA and did not exhibit a power-law form i.e., not a straight line on the log-log plot (Figure 2, Text S4 and Figure S3). This loss of the power-law form can be visualized by quantifying the local slope of *F*(*n*), which gradually increased from ∼0.5 at time scales <0.05 hours to ∼1.5 at 0.6–1.5 hours, and to even larger values at larger time scales (Figure 2). The non-power-law form of *in vitro F*(*n*), that was virtually identical in all individual mice and rats, indicates absence of fractal patterns in *in vitro* MUA fluctuations.

To check the stability of the *in vitro F*(*n*) from the beginning to the end of the recordings, we performed detrended fluctuation analyses in non-overlapping 12-hour windows for each recording. We found that the *F*(*n*) of each 12-hour window was similar to that of the whole *in vitro* recordings (Figure S4). Since *in vitro* recordings were started ∼1 hour after harvesting the SCN, this result presumably indicates that loss of fractal MUA fluctuations occurred as soon as the SCN was isolated from the body.

**Figure 3:**
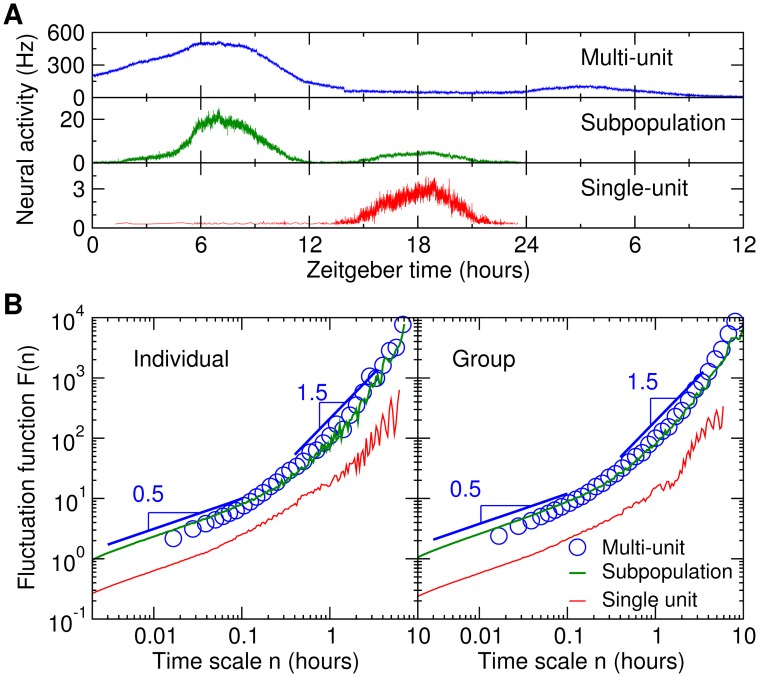
Single unit and subpopulation neural activity of *in vitro* SCN possess the same non-fractal fluctuation patterns as observed in *in vitro* multi-unit activity. (**A**) Examples of single unit, subpopulation, and multi-unit neural activity within the *in vitro* SCN of the same mouse. The recordings were selected to reflect the possibility that single-unit, subpopulation, and multi-unit data could show different circadian profiles as described before [18], e.g., the single-unit data peaked at Zeitgeber time 18 hours; the subpopulation data peaked at 7 and 18 hours, respectively; and the multi-unit activity peaked at 7 hours and 3 hours, respectively. (**B**) The fluctuation functions of the individual recordings (shown in panel A), and the group averages. Data were shown in log-log plots. The form of the function was almost identical for single-unit and subpopulation data as well as for the MUA recordings except for a vertical shift which indicates an expected difference in mean fluctuation amplitude. The subpopulation data were obtained from the analysis of action potentials with the target average firing rate of 10 Hz in the 30-minute windows centered at the peaks of MUA (see Methods). The form remained the same for different subpopulation data with different target average firing rate at MUA peak(s). The multi-unit results were vertically shifted for a better visualization of the similar non-power-law form as compared to single-unit and subpopulation results.

We further examined in mice whether the absence of a fractal pattern in MUA of *in vitro* SCN also occurred in much smaller subpopulations of *in vitro* SCN neurons as well as individual *in vitro* SCN neurons (Figure 3A). We found that the shape of the *in vitro F*(*n*) was independent of the number of *in vitro* SCN neurons selected (see Methods), i.e., the *F*(*n*) had the same non-power law form in these subpopulations and individual SCN neurons (Figure 3). The non-power law F(n) was virtually identical to that for MUA across the scale range of ∼0.02–5 hours. At very small time scales (∼0.002–0.02 hours; or ∼7–70 seconds) the *F*(*n*) of subpopulations and individual SCN neurons exhibited consistent behavior with the local slope converging to 0.5 (indicating random noise). Thus, in this viable *in vitro* preparation in which overall circadian rhythmicity in mean neural activity level persists, individual SCN neurons, small subpopulations of neurons and larger groups of neurons do not possess fractal activity patterns at time scales >0.002 hours (Figure 3 and Figure S4).

## Discussion

Motor activity fluctuations in both humans and rodents display robust fractal temporal structures [2], [8], which require a network of feedback interactions among control nodes operating at different time scales [22], [23]. We previously found that the SCN plays an essential role in normal fractal control of motor activity at time scales from minutes up to 24 hours [8]. In this study we found that the neural activity of the SCN itself displays fractal fluctuations over a range of time scales from ∼0.02 up to 10 hours. These fractal patterns were almost identical between mice and rats. Moreover, the fractal SCN activity persisted when the light-induced afferent input to the SCN was eliminated during constant dark conditions. These findings indicate the existence of long-range fractal regulation in SCN neural activity. However, the patterns completely broke down when the SCN was isolated from the body even though the *in vitro* SCN continued to exhibit circadian oscillations in mean levels of MUA. Thus, it appears that fractal patterns at time scales >0.02 hours emerge from the interplay between the SCN and extra-SCN areas.

### Physiological significances of fractal patterns

Many physiological processes and neural dynamics exhibit fractal regulation generating complex fluctuations that display similar, strong correlations across a wide range of time scales [4], [24]. Based on theoretical models in physics, it has been hypothesized that fractal correlations indicate the existence of a system at, or near, a “critical state” [23]. Theoretically, a system under such a critical state, perched between different stable states, is optimally prepared to respond to intrinsic/extrinsic influences by orchestrating subunits within the system in a coherent manner. This hypothesis is appealing because it bestows upon the fractal phenomenon a physiological meaning related to system integrity and adaptability. Numerous studies support this hypothesis, showing, for instance, that complexity and fractal patterns of physiological fluctuations: (i) are reduced with diseases and aging [1], [5]; (ii) are correlated with adaptability under challenging conditions [25]; and (iii) can predict treatment outcome of patients with ventricular fibrillation [26] and can predict mortality of patients after stroke [6]. Thus, it is tempting to infer that the robust fractal pattern in the *in vivo* SCN neural activity and the complete breakdown of this pattern in the *in vitro* SCN reflect a vital, multiscale control function of the SCN in the intact organism.

### Complex SCN neural interactions

Despite clear evidence of its importance to biology, fractal control in physiological systems still defies understanding based on traditional mechanistic models. Mathematical models predict that fractal fluctuations require an integrated network of multiple control nodes with feedback interactions [27]. However, there have been few studies attempting to identify neural circuitry (neural nodes and pathways) for fractal physiological regulation. Currently, the SCN is the only neural node that has been shown to impact fractal regulation in physiological functions [8] [28]. Thus, a natural choice for the exploration of the fractal control network is to investigate the SCN-related neural interactions.

The neural network within the SCN is composed of tens of thousands of coupled neurons each acting as a cell-autonomous oscillator [29]. Based on differences in transmitter content, a distinction has been made between a core region, containing vasoactive intensital popypeptide, and a shell region, containing vasopressin [30]. Other neurotransmitters include calretinin, neurotensin, gastrin releasing peptide, arginine vasopressin, angiotensin II and met-enkephalines [31]. Communication mechanisms, including chemical synaptic transmission and gap junctions, regulate the degree of phase heterogeneity observed among SCN neurons [32]. It is intriguing that such a complex biological network is unable to produce fractal patterns from ∼0.02 up to 10 hours. Our findings indicate that the fractal patterns of the SCN neural activity and motor activity at >0.02 hours require feedback interactions between the SCN and other control nodes in a larger network, i.e. the neural network within the SCN is only a part of the fractal control network. It is yet to be clarified whether the SCN is only a crucial mediator that relays fractal regulatory information generated from other nodes to various efferent nodes, or the neural interactions between the SCN and other nodes are the key component generating fractal fluctuations in neurophysiological functions. Conceivably, besides the SCN, this fractal control network could incorporate: input to the SCN from other neuronal sites (e.g., intergeniculate leaflet, midbrain raphe, paraventricular thalamus, limbic telencephalon, and pedunculopontine/laterodorsal tegmental nuclei) [15], [17], [33]–[35]; direct and indirect efferent pathways from the SCN (e.g., to the medial preoptic region, subparaventricular zone, paraventricular nucleus, lateral hypothalamus, ventrolateral preoptic nucleus and dorsomedial nucleus of the hypothalamus); humoral factors secreted by the SCN (e.g., transforming growth factor α and prokineticin 2) [36]–[39] ; and humoral factors influencing SCN function. Potential future approaches that may help determine which of these SCN interactions are part of the fractal control network include: (i) infusion of tetrodotoxin into the SCN to temporarily block the afferent and efferent pathways without affecting intrinsic SCN oscillation [40]; (ii) SCN transplantation to explore the separate neural and humoral interactions between the SCN and extra-SCN nodes [41]; (iii) targeted neural lesions of potential nodes in the fractal control network; and (iv) gene manipulations (e.g. Bmal1 and Clock knockouts [36], [37]) to determine the molecular components of these fractal regulations.

### Fractal regulation in different ranges of time scales

Two groups previously examined the SCN *in vitro* and reported a fractal pattern in firing rate of individual SCN neurons [42], [43], which seemingly opposes our finding. We note that the tested ranges of time scales in the previous studies (3 to 9 seconds and ∼5 to 500 seconds, respectively) are different from our study (60 to ∼18,000 seconds for MUA and 6 to ∼5,400 seconds for single unit and subpopulation activity). One plausible explanation is that maintenance of the fractal neural activity patterns at different time scales requires different control nodes, pathways, and interactions, especially at time scales <6 seconds where we could not examine fractal properties due to limited sampling resolution of our data (Text S5). Alternatively, limitations related to previous use and/or interpretation of fractal analysis may also lead to incorrect detection of fractal patterns (see more discussion in Text S5). In this study, we examined fractal patterns using the detrended fluctuation analysis which can better identify long-range fractal correlations while avoiding spurious detection of apparent fractal patterns that are an artifact of nonstationarity in signals [1], [2], [5]. The observed fractal patterns in the *in vivo* SCN neural activity and non-fractal patterns in the *in vitro* SCN neural activity were very robust at all tested time scales from ∼0.02 up to 10 hours — the range over which many physiological variables such as heart rate and motor activity also display fractal fluctuations under healthy conditions [1], [2]. Thus, feedback interactions between SCN and extra-SCN tissue are likely essential for generating/maintaining fractal regulation in overt physiological functions over such a wide range of time scales. It is worth noting that fractal patterns at smaller time scales may not necessarily require the same feedback interactions and further studies are warranted to address the matter.

### Limitations

In this study we analyzed *in vivo* and *in vitro* MUA recordings of the SCN that were collected from previous experiments to assess circadian rhythmicity of SCN neural activity. Continuous and long recordings are required for reliable assessment of fractal properties over the selected wide range of time scales (at least up to 5 hours) [20]. Thus, we had to exclude recordings that are either too short (<36 hours) or too fragmented due to missing/contaminated data points. For the same reason, we could only examine the fractal patterns at time scales up to 10 hours. We also excluded MUA recordings with significant behaviorally-induced suppressions [15] because the current study is focused on fractal regulation in spontaneous fluctuations of the SCN neural activity. As a result, the sample size in this study is relatively small.

Moreover, one potential concern regarding the *in vitro* SCN activity would be whether the specific SCN slice preparations used in the current study would cause disturbances of neural connections within the SCN network that would abolish fractal patterns in the SCN. If this would be the case, we would expect that the fractal patterns would have been less disturbed in slices that contain a larger proportion of the SCN. However, all individual *in vitro* recordings showed the same consistent scaling characteristics (non-fractal), independent of the extent of the SCN *in vitro* (Figure S5). Moreover, the non-fractal pattern was the same for subpopulation and single unit data (Figure 3), and was independent of the electrode location within the *in vitro* SCN (see Table S1; and Figure S5). Thus, it is unlikely that lack of fractal pattern in the *in vitro* SCN activity at tested time scales from ∼6–18,000 seconds was caused by disrupted integrity of the within-SCN neural network or the recording technique. However, in order to formally address the concern, different experimental approaches such as *in situ* investigations are needed, e.g. blocking the input and output pathways of the SCN without affecting the neural connections within the SCN.

### Future directions

A major challenge to neuroscience and circadian biology is to understand how the circadian system orchestrates its repertoire of adaptive physiological and behavioral functions and how disruption of this circadian control may contribute to variation in disease susceptibility. Our data indicate that the SCN is involved in setting a temporal program in a broader sense than has previously been appreciated. Not only is the SCN involved in generating 24-hour rhythms, but in its interaction with extra-SCN areas, fractal patterns in SCN activity are generated across a broad range of time scales from ∼0.02 to10 hours. Our results raise the importance of studying and understanding the interactions between the SCN and the other elements that together are responsible for these fractal patterns. Moreover, it seems likely that that these fractal patterns within SCN neural activity *in vivo* are transmitted to fractal patterns in physiological and behavioral function. For instance, by studying SCN-lesioned animals, we previously found that the SCN is required for fractal regulation of motor activity [8] and cardiac function [28], and it seems plausible that the SCN could be involved in the fractal control of many other facets of physiology and behavior, including brain function and sleep patterns [15]. For a better understanding of the multiscale regulatory function of the circadian network and its relevance to system adaptability, it is important to identify other control nodes (other than the SCN) and their interactions with the SCN that are involved in fractal neurophysiological regulation. Finally, there is accumulating evidence that normal function of the circadian system is vital for health and that impaired circadian function leads to disorders of diverse physiological processes [36], [37]. Thus, it is important to determine whether chronic disturbance of the circadian system, as occurs with shift work, also affects fractal regulation of neurophysiological functions, and whether the loss of fractal function leads to malfunction.

## Supporting Information

Figure S1 **Fractal correlations of motor activity fluctuations in mice.** (**A**) Motor activity recordings of a representative mouse during the light/dark (LD) cycles and during constant darkness (DD). (**B**) The fluctuation functions of the signals shown in Panel A. (C) The group average of the fluctuation function obtained from 5 mice. Scaling exponent α = 0.91±0.01 (SE) during light/dark (LD) cycles and 0.92±0.01 (SE) during constant darkness (DD).
(DOC)

Figure S2 **Similar fractal patterns in the SCN neural activity during the light and dark phases of the light-dark (LD) cycles.** (**A**) Multi-unit activity (MUA) of the *in vivo* SCN collected from a mouse during light-dark cycles (the same signal shown in **Figure 1A**). (**B**) Detrended fluctuation function F(n) of the MUA recordings shown in A during the light phase (open circles) and during the dark phase (filled circles). We found similar fractal patterns in the two phases (**Figure S2**), as characterized by a similar scaling exponent during the dark phase (group mean ± SE; mice: 1.02±0.03; rats: 1.09±0.05) and during the light phase (mice: 1.04±0.03; rats: 1.05±0.04; p = 0.18).
(DOC)

Figure S3 **Deviation of the fluctuation function, F(n), from power-law fit.** (**A**) Fluctuation functions of two individual mice (one for *in vivo* and one for *in vitro* recordings) and two rats (one for *in vivo* and one for *in vitro* recordings). The black solid line is the power-law fit for the *in vivo* mouse data and the red dashed line is for the *in vitro* mouse data. The scaling curves were vertically shifted to better visualize the similar functional form between mice and rats. (**B**) % of deviation of F(n) from power-law fit at different time scales. Results were obtained from data shown in Panel A. (**C**) Total % of points (uniformly distributed in log scale) with deviations greater than a specified percentage. Power-law fit was obtained at time scales from ∼0.02–5 hours. Clearly, the power-law fit of the *in vitro* data was erroneous, leading to large deviation of the original F(n) at almost all time scales.
(DOC)

Figure S4 **Detrended fluctuation function of** ***in vitro*** **MUA during different 12-hour periods.** The *in vitro* MUA recording was ∼40 hours in duration (shown in **Figure 1A**) and started ∼1 hour after harvesting the SCN. The fluctuation function *F*(*n*) was similar for all 12-hour periods. Shuffling MUA data destroyed the correlations in the signal, leading to a white-noise type of fluctuation that is characterized by a power-law *F*(*n*) with a scaling exponent = 0.5.
(DOC)

Figure S5 **The non-fractal fluctuation pattern of the** ***in vitro*** **SCN activity is independent of the size of the SCN slice.** Shown are the detrended fluctuation functions of 3 SCN slices that contained 90%, 70% and 40% of the SCN in the rostro-caudal plane, respectively (mouse 6, 4, and 3 in Table S1, respectively). The scaling curves were vertically shifted to better visualize the similar functional form of the *in vitro* results. In addition, the three *in vitro* recordings were collected from the anterior, medial, and posterior part of the SCN, respectively. As comparison, the group average of the fluctuation functions of the *in vivo* SCN activity is also presented.
(DOC)

Text S1 **Effects of missing data and down-sampling on the detrended fluctuation analysis.**
(DOC)

Text S2 **Fractal patterns of motor activity in mice.**
(DOC)

Text S3 **Persistent circadian rhythmicity and reduced ultradian fluctuations in the** ***in vitro*** **SCN neural activity.**
(DOC)

Text S4 **Testing of power-law form.**
(DOC)

Text S5 **Fractal or non-fractal fluctuations in the** ***in vitro*** **SCN neural activity?**
(DOC)

Table S1 **Information of SCN slices and corresponding** ***in vitro*** **recordings from 7 mice.**
(DOC)
