# Supplementary material for: Fractal Patterns of Neural Activity Exist within the Suprachiasmatic Nucleus and Require Extrinsic Network Interactions
Source: PLoS One. 2012 Nov 20;7(11):e48927. doi: 10.1371/journal.pone.0048927 (PMC3502397; doi:10.1371/journal.pone.0048927)
Supplement: Figure S1 — Fractal correlations of motor activity fluctuations in mice. (A) Motor activity recordings of a representative mouse during the light/dark (LD) cycles and during constant darkness (DD). (B) The fluctuation functions of the signals shown in Panel A. (C) The group average of the fluctuation function obtained from 5 mice. Scaling exponent α = 0.91±0.01 (SE) during light/dark (LD) cycles and 0.92±0.01 (SE) during constant darkness (DD). (DOC) [file pone.0048927.s001.doc]

|  |
| --- |
| **Figure S1.** Fractal correlations of motor activity fluctuations in mice. (A) Motor activity recordings of a representative mouse during the light/dark (LD) cycles and during constant darkness (DD). (B) The fluctuation functions of the signals shown in Panel A. (C) The group average of the fluctuation function obtained from 5 mice. Scaling exponent α = 0.91± 0.01 (SE) during light/dark (LD) cycles and 0.92 ± 0.01 (SE) during constant darkness (DD). |
