# Supplementary material for: Fractal Patterns of Neural Activity Exist within the Suprachiasmatic Nucleus and Require Extrinsic Network Interactions
Source: PLoS One. 2012 Nov 20;7(11):e48927. doi: 10.1371/journal.pone.0048927 (PMC3502397; doi:10.1371/journal.pone.0048927)
Supplement: Figure S2 — Similar fractal patterns in the SCN neural activity during the light and dark phases of the light-dark (LD) cycles. (A) Multi-unit activity (MUA) of the in vivo SCN collected from a mouse during light-dark cycles (the same signal shown in Figure 1A ). (B) Detrended fluctuation function F(n) of the MUA recordings shown in A during the light phase (open circles) and during the dark phase (filled circles). We found similar fractal patterns in the two phases (Figure S2), as characterized by a similar scaling exponent during the dark phase (group mean ± SE; mice: 1.02±0.03; rats: 1.09±0.05) and during the light phase (mice: 1.04±0.03; rats: 1.05±0.04; p = 0.18). (DOC) [file pone.0048927.s002.doc]

|  |
| --- |
| **Figure S2.** Similar fractal patterns in the SCN neural activity during the light and dark phases of the light-dark (LD) cycles. (A) Multi-unit activity (MUA) of the *in vivo* SCN collected from a mouse during light-dark cycles (the same signal shown in **Figure 1A**). (B) Detrended fluctuation function F(n) of the MUA recordings shown in A during the light phase (open circles) and during the dark phase (filled circles). We found similar fractal patterns in the two phases (**Figure S2**), as characterized by a similar scaling exponent during the dark phase (group mean ± SE; mice: 1.02 ± 0.03; rats: 1.09 ± 0.05) and during the light phase (mice: 1.04 ± 0.03; rats: 1.05 ± 0.04; p=0.18). |
