# Supplementary material for: Fractal Patterns of Neural Activity Exist within the Suprachiasmatic Nucleus and Require Extrinsic Network Interactions
Source: PLoS One. 2012 Nov 20;7(11):e48927. doi: 10.1371/journal.pone.0048927 (PMC3502397; doi:10.1371/journal.pone.0048927)
Supplement: Figure S3 — Deviation of the fluctuation function, F(n), from power-law fit. (A) Fluctuation functions of two individual mice (one for in vivo and one for in vitro recordings) and two rats (one for in vivo and one for in vitro recordings). The black solid line is the power-law fit for the in vivo mouse data and the red dashed line is for the in vitro mouse data. The scaling curves were vertically shifted to better visualize the similar functional form between mice and rats. (B) % of deviation of F(n) from power-law fit at different time scales. Results were obtained from data shown in Panel A. (C) Total % of points (uniformly distributed in log scale) with deviations greater than a specified percentage. Power-law fit was obtained at time scales from ∼0.02–5 hours. Clearly, the power-law fit of the in vitro data was erroneous, leading to large deviation of the original F(n) at almost all time scales. (DOC) [file pone.0048927.s003.doc]

|  |
| --- |
| **Figure S3.** Deviation of the fluctuation function, F(n), from power-law fit. (**A**) Fluctuation functions of two individual mice (one for *in vivo* and one for *in vitro* recordings) and two rats (one for *in vivo* and one for *in vitro* recordings). The black solid line is the power-law fit for the *in vivo* mouse data and the red dashed line is for the *in vitro* mouse data. The scaling curves were vertically shifted to better visualize the similar functional form between mice and rats. (**B**) % of deviation of F(n) from power-law fit at different time scales. Results were obtained from data shown in Panel A. (**C**) Total % of points (uniformly distributed in log scale) with deviations greater than a specified percentage. Power-law fit was obtained at time scales from ~0.02 - 5 hours. Clearly, the power-law fit of the *in vitro* data was erroneous, leading to large deviation of the original F(n) at almost all time scales. |
