# Supplementary material for: Fractal Patterns of Neural Activity Exist within the Suprachiasmatic Nucleus and Require Extrinsic Network Interactions
Source: PLoS One. 2012 Nov 20;7(11):e48927. doi: 10.1371/journal.pone.0048927 (PMC3502397; doi:10.1371/journal.pone.0048927)
Supplement: Figure S4 — Detrended fluctuation function of in vitro MUA during different 12-hour periods. The in vitro MUA recording was ∼40 hours in duration (shown in Figure 1A ) and started ∼1 hour after harvesting the SCN. The fluctuation function F(n) was similar for all 12-hour periods. Shuffling MUA data destroyed the correlations in the signal, leading to a white-noise type of fluctuation that is characterized by a power-law F(n) with a scaling exponent = 0.5. (DOC) [file pone.0048927.s004.doc]

|  |
| --- |
| **Figure S4.** Detrended fluctuation function of *in vitro* MUA during different 12-hour periods. The *in vitro* MUA recording was ~40 hours in duration (shown in **Figure 1A**) and started ~1 hour after harvesting the SCN. The fluctuation function *F*(*n*) was similar for all 12-hour periods. Shuffling MUA data destroyed the correlations in the signal, leading to a white-noise type of fluctuation that is characterized by a power-law *F*(*n*) with a scaling exponent = 0.5. |
