# Supplementary material for: Fractal Patterns of Neural Activity Exist within the Suprachiasmatic Nucleus and Require Extrinsic Network Interactions
Source: PLoS One. 2012 Nov 20;7(11):e48927. doi: 10.1371/journal.pone.0048927 (PMC3502397; doi:10.1371/journal.pone.0048927)
Supplement: Figure S5 — The non-fractal fluctuation pattern of the in vitro SCN activity is independent of the size of the SCN slice. Shown are the detrended fluctuation functions of 3 SCN slices that contained 90%, 70% and 40% of the SCN in the rostro-caudal plane, respectively (mouse 6, 4, and 3 in Table S1, respectively). The scaling curves were vertically shifted to better visualize the similar functional form of the in vitro results. In addition, the three in vitro recordings were collected from the anterior, medial, and posterior part of the SCN, respectively. As comparison, the group average of the fluctuation functions of the in vivo SCN activity is also presented. (DOC) [file pone.0048927.s005.doc]

|  |
| --- |
| **Figure S5**. The non-fractal fluctuation pattern of the *in vitro* SCN activity is independent of the size of the SCN slice. Shown are the detrended fluctuation functions of 3 SCN slices that contained 90%, 70% and 40% of the SCN in the rostro-caudal plane, respectively (mouse 6, 4, and 3 in Table S1, respectively). The scaling curves were vertically shifted to better visualize the similar functional form of the *in vitro* results. In addition, the three *in vitro* recordings were collected from the anterior, medial, and posterior part of the SCN, respectively. As comparison, the group average of the fluctuation functions of the *in vivo* SCN activity is also presented. |
