# Supplementary material for: Fractal Patterns of Neural Activity Exist within the Suprachiasmatic Nucleus and Require Extrinsic Network Interactions
Source: PLoS One. 2012 Nov 20;7(11):e48927. doi: 10.1371/journal.pone.0048927 (PMC3502397; doi:10.1371/journal.pone.0048927)
Supplement: Text S1 — Effects of missing data and down-sampling on the detrended fluctuation analysis. (DOC) [file pone.0048927.s006.doc]

**Down-sampling**

In this study, there were 7 *in vivo* MUA recordings with missing data points or segments every few minutes, likely due to movement artifacts. In order to assess fractal patterns, we down-sampled these recordings using the following procedure: a raw MUA recording (epoch length = 10 seconds) was first divided into non-overlapping 600-second windows, and in each window (60 points), average MUA was obtained from all non-missing values.

**Effects of missing data and down-sampling on the detrended fluctuation analysis**

To estimate possible effects of missing data and the down-sampling procedure (see above), we applied the characteristics of each down-sampled MUA signal to 10 *in* *vivo* MUA recordings of mice that contained no or very little missing data (<.0006% in all cases). First we obtained 10 control signals by trimming the 10 mouse recordings to the same length as the raw data of the down-sampled MUA signal. Then we generated surrogate signals from control signals by: (1) locating the positions of missing data points in the raw data of the to-be down-sampled signal; (2) deleting data points in control signals at those positions; and (3) applying the down-sampling procedure to the control signals with artificially introduced missing data points. Finally, we applied the detrended fluctuation analysis (DFA) to the control signals and to the surrogate signals. Thus, for each down-sampled MUA recording, we obtained 10 scaling exponents from the control signals — “control exponents” — and 10 from the surrogate signals — “treated exponents”. Note that the two types of exponents were obtained over different time scale ranges (control exponents: from ~1 minute to >=5 hours; treated exponents: from ~1 hour to >=5 hours) due to the different time scale resolutions of the signals (i.e., an epoch length of 10 seconds for control signals, and of 600 seconds for surrogate signals).

To determine whether there was a significant effect of missing data and down-sampling, we compared control exponents and treated exponents using a linear model with each mouse recording as a random effect. If the effect was significant, we adjusted the scaling exponent of the down-sampled MUA signal by the average difference between control and treated exponents. In 5 of the 7 down-sampled MUA recordings, we found significant differences between control and treated exponents. The difference varied (range: -0.082 to 0.193; Mean ± SE: 0.035 ± 0.040), possibly due to the different amount and distribution of missing data points.
