# Supplementary material for: Fractal Patterns of Neural Activity Exist within the Suprachiasmatic Nucleus and Require Extrinsic Network Interactions
Source: PLoS One. 2012 Nov 20;7(11):e48927. doi: 10.1371/journal.pone.0048927 (PMC3502397; doi:10.1371/journal.pone.0048927)
Supplement: Text S2 — Fractal patterns of motor activity in mice. (DOC) [file pone.0048927.s007.doc]

**Fractal patterns of motor activity in mice**

We have previously observed similar fractal patterns in motor activity between humans and rats [1,2]. To test whether motor activity fluctuations in mice also possess fractal patterns, we analyzed motor activity of 5 free-moving mice that were simultaneously collected with neural activity of the *in vivo* SCN (see **Methods**). The fluctuation function displayed a power-law form over a range of time scales from ~1 minute to at least 5 hours (**Figure S1**). The scaling exponent α ~0.9 indicated strong fractal correlations in the fluctuations that remained the same during light-dark cycles (mean ± SE: 0.91 ± 0.01) and during constant darkness (0.92 ± 0.01). These fractal correlations are similar to those previously observed in motor activity of humans and rats [1,2]. Compared to the SCN neural activity from the same animals, the scaling exponent of motor activity was slightly but significantly smaller (mixed model ANOVA p = 0.023). Such a slight difference might suggest that fractal fluctuations of the SCN neural activity are not passively transduced into fractal patterns in motor activity.

References:

1. Hu K, Ivanov PC, Chen Z, Hilton MF, Stanley HE, Shea SA (2004) Non-random fluctuations and multi-scale dynamics regulation of human activity. Physica A 337: 307-318.

2. Hu K, Scheer FA, Ivanov PC, Buijs RM, Shea SA (2007) The suprachiasmatic nucleus functions beyond circadian rhythm generation. Neuroscience 149: 508-517.
