# Supplementary material for: Fractal Patterns of Neural Activity Exist within the Suprachiasmatic Nucleus and Require Extrinsic Network Interactions
Source: PLoS One. 2012 Nov 20;7(11):e48927. doi: 10.1371/journal.pone.0048927 (PMC3502397; doi:10.1371/journal.pone.0048927)
Supplement: Text S3 — Persistent circadian rhythmicity and reduced ultradian fluctuations in the in vitro SCN neural activity. (DOC) [file pone.0048927.s008.doc]

**Persistent circadian rhythmicity and reduced ultradian fluctuations in the *in vitro* SCN neural activity**

To verify the viability of the *in vitro* SCN cells, we examined the rhythmicity of *in vitro* MUA (see Methods)[1]. Overall, circadian rhythms were more pronounced while the variation in the ultradian region was reduced in the *in vitro* MUA as compared to *in vivo* MUA (**Figure 1** and **Figure 3**). The normalized peak-to-trough amplitude of the *in vitro* MUA (relative to the variation level at ultradian time scales) was 33.5 ± 6.5 (mean ± SE) for mice and 28.8 ± 2.7 for rats, which was much larger than the values of the *in vivo* MUA (mice: 3.0 ± 0.4; rats 2.9 ± 1.6; mixed ANOVA p <0.0001).

Additionally, the circadian period of *in vitro* MUA was significantly shorter than 24 hours in both species (median ± SE; mice: 21.0 ± 1.2 hours; rats: 22.0 ± 0.8 hours; p = 0.0014) and significantly shorter than the period of *in vivo* MUA during DD (mice: 24.0 ± 1.0 hours; rats: 23.8 ± 0.5 hours; ANOVA p = 0.026). Small but significant differences between behavioral and *in vitro* periodicities have been previously reported [2,3].

References

1. Huang NE, Shen Z, Long SR, Wu MC, Shih EH, Zheng Q, Tung CC, Liu HH (1998) The empirical mode decomposition method and the Hilbert spectrum for non-stationary time series analysis. Proc Roy Soc London A454: 903-995.

2. Pendergast JS, Friday RC, Yamazaki S (2009) Endogenous rhythms in Period1 mutant suprachiasmatic nuclei in vitro do not represent circadian behavior. J Neurosci 29: 14681-14686.

3. Noguchi T, Watanabe K (2008) Regional differences in circadian period within the suprachiasmatic nucleus. Brain Res 1239: 119-126.
