# Supplementary material for: Fractal Patterns of Neural Activity Exist within the Suprachiasmatic Nucleus and Require Extrinsic Network Interactions
Source: PLoS One. 2012 Nov 20;7(11):e48927. doi: 10.1371/journal.pone.0048927 (PMC3502397; doi:10.1371/journal.pone.0048927)
Supplement: Text S4 — Testing of power-law form. (DOC) [file pone.0048927.s009.doc]

**Testing of power-law form**

To quantify how well a fluctuation function F(n) obeys power-law form, we examined the percentage deviation of F(n) from its power-law fit P(n) at different time scales (0.02-5 hours), i.e., % deviation is equal to the absolute value of F(n)/P(n)-100%. Sampled data points in F(n) were uniformly distributed in the log scale (~8 points in an interval of log2). The average % deviation of *in vivo* F(n) from power-law fit was 5.5 ± 0.7% (SE) for mice and 9.1 ± 1.9% for rats, which was much smaller than that of *in vitro* F(n) (mice: 37 ± 2%; rats: 32 ± 3%; p<0.0001; no interaction effect regarding species) The large deviation of *in vitro* F(n) from power-law fit was not due to randomly distributed “outliers” in F(n) (**Figure S3**). Instead, in each *in vitro* F(n), there were more than 44% of sampled points with deviation >20% from the power-law fit and these points distributed across the whole fit range of time scales. Such large deviation of F(n) from power-law fit at almost all tested time scales is a mathematical indication that *in vitro* MUA fluctuations are not fractal.
