# Supplementary material for: Fractal Patterns of Neural Activity Exist within the Suprachiasmatic Nucleus and Require Extrinsic Network Interactions
Source: PLoS One. 2012 Nov 20;7(11):e48927. doi: 10.1371/journal.pone.0048927 (PMC3502397; doi:10.1371/journal.pone.0048927)
Supplement: Text S5 — Fractal or non-fractal fluctuations in the in vitro SCN neural activity? (DOC) [file pone.0048927.s010.doc]

**Fractal or non-fractal fluctuations in the *in vitro* SCN neural activity?**

We found in this study that the neural activity of the *in vitro* SCN exhibited non-fractal fluctuations while two previous studies reported fractal properties in the firing rate of individual *in vitro* SCN neurons [1,2]. This discrepancy could be caused by differences in the study design such as the method and the range of time scale for the assessment of fractal properties, as well as certain debatable result interpretations (see below).

First, activity data of individual SCN neurons were collected from rats in the previous studies and from mice in this study. It is yet to be determined whether the inconsistent results reflect a difference between two species. But this possibility is highly unlikely
because the fluctuation patterns were identical between mice and rats for *in vitro* MUA, for *in vivo* MUA, and even for motor activity patterns (see Figure 2 and Figure S1).

Second, the thickness of the SCN slices was 120-150 μm in the study of Kim et al., was 500 μm in the study of Brown et al., and varied over a wide range in this study (i.e., containing 40%-90% of SCN; Table S1). The thickness of the SCN slices is different in each of the three studies though all slices were cut along the coronal plane in all studies. For instance, Such difference in slice preparations may have different impacts on the neural connections within the SCN network, leading to different alterations in the fluctuation patterns of *in vitro* SCN neural activity. However, this possibility seems also unlikely because we found the same scaling characteristic (non-fractal) for different SCN slices that contained very different amount of SCN *in vitro* (Figure S5).

Third, to examine fractal correlations in the SCN activity, both previous studies calculated the Fano factor (FF) at different time scales [1,2]. However, simulation results provided clear evidence that, for a signal with fractal correlations, the FF analysis significantly underestimates the fractal correlations and the degree of such a bias depends on the length and the fractal properties of data [3]. For instance, for a fractal correlated signal with one million data points and an assigned FF scaling exponent α_F_ =0.8 (corresponding to the DFA α of 0.9; α_F_ = 2α - 1), the estimated α_F_ could be as low as 0.47 (α = 0.74). More importantly, due to mathematical constraints, the local slope of the FF function in the log-log plot cannot be greater than 1. Thus, the FF function can falsely suggest fractal correlations, for instance, in a non-fractal signals that have a DFA function with the local slope much greater than 1. How these limitations of the FF affected the previous results and their interpretation justifies further examination.

Recognizing the problems of the FF, Kim et al. also adopted two other fractal analyses, namely, the Allan factor (AF) and the periodogram (PG) [1], which had relatively better performance than the FF [4]. Based on the exponent of the AF (corresponding to DFA α = 0.91 ± 0.19; mean±SD) and PG (corresponding to DFA α = 0.93 ± 0.18), Kim et al. concluded that each SCN neuron possesses fractal neural activity with positive correlations in the fluctuations. However, it is important to note that the existence of fractal correlations should not be only based on the fitting exponent (e.g., DFA α >0.5) but also requires a power-law form of the fluctuation function (see Figure 2, Figure S3 and Text S3). Forcing a power-law fit to a non-power-law function, without a wide enough range of time scales to test scale-invariance and/or without testing if the power-law fit is appropriate, can lead to an incorrect conclusion that there exists a fractal pattern. For instance, for the current data sets, a power-law fit of the *in vitro* fluctuation function *F*(*n*) at time scales from 60-7200 seconds would yield an exponent of ~ 1.15 (see the red dashed line in Figure S3A) which is close to the scaling exponent of the *in vivo* MUA although this fit would not be valid because the *in vitro* *F*(*n*) is clearly non-power-law over the time scale range. A careful examination of Figure 2A and 2C in Ref [1] revealed that the AF and PG functions presented by Kim et al. did not obey a power-law form in the fitting range (AF: 5-200 seconds; PG: ~0.003-0.1 Hz) where the exponent was calculated. Thus, what Kim et al. observed appears to be non-fractal correlations, in agreement with our study (Figure 2, Figure S4, and Figure S5).

Finally, the time scale range we tested fractal properties was 60 to ~18,000 seconds for MUA and 6 to ~5,400 seconds for single unit and subpopulation activity. This is quite different from the study of Brown et al. in which the FF exponent (α_F_ ranging from 0.20-0.4; corresponding to the DFA α from 0.6-0.7) was obtained for a time scale range of 3-9 seconds. In such a narrow range of time scales, it is still a challenge to determine whether or not a function obeys a power-low form [5]. Based on our results of single unit and subpopulation activity recordings over a wider range of time scales (6 to ~5,400 seconds), it seems that the DFA-derived fluctuation function does not obey a power-law, indicating non-fractal fluctuations (Figure 3). However, we could not exclude the possibility that fractal regulation with weak correlations (e.g., the DFA α = 0.6-0.7) at very small time scales (<6 seconds) may be present within individual SCN cells while fractal patterns at larger time scales (>6 seconds) may require interactions between the SCN and other neuronal nodes. Note, that such a possibility does not necessarily indicate that normal functionality of individual SCN neurons is not important for the fractal patterns at large time scales (i.e., it may be essential for the fractal patterns at all time scales).

References

1. Kim SI, Jeong J, Kwak Y, Kim YI, Jung SH, Lee KJ (2005) Fractal stochastic modeling of spiking activity in suprachiasmatic nucleus neurons. J Comput Neurosci 19: 39-51.

2. Brown TM, Coogan AN, Cutler DJ, Hughes AT, Piggins HD (2008) Electrophysiological actions of orexins on rat suprachiasmatic neurons in vitro. Neuroscience Letters 448: 273-278.

3. Lowen SB, Teich MC (1995) Estimation and Simulation of Fractal Stochastic Point-Processes. Fractals-An Interdisciplinary Journal on the Complex Geometry of Nature 3: 183-210.

4. Lowen SB, Teich MC (1996) The periodogram and Allan variance reveal fractal exponents greater than unity in auditory-nerve spike trains. J Acoust Soc Am 99: 3585-3591.

5. Chu-Shore J, Westover MB, Bianchi MT (2010) Power law versus exponential state transition dynamics: application to sleep-wake architecture. PLoS ONE 5: e14204.
