# Supplementary material for: Fractal Patterns of Neural Activity Exist within the Suprachiasmatic Nucleus and Require Extrinsic Network Interactions
Source: PLoS One. 2012 Nov 20;7(11):e48927. doi: 10.1371/journal.pone.0048927 (PMC3502397; doi:10.1371/journal.pone.0048927)
Supplement: Table S1 — Information of SCN slices and corresponding in vitro recordings from 7 mice. (DOC) [file pone.0048927.s011.doc]

**Table S1. Information of SCN slices and corresponding *in vitro* recordings from 7 mice.**

| **Mouse** | **Section 1** | **Section 2** | **Total amount of the SCN** | **Electrode position** | **Recording duration (hours)** |
| --- | --- | --- | --- | --- | --- |
| 1* | 40% | 100% | 60% | Medial | 40 |
| 2* | 20% | 100% | 80% | Anterior | 36 |
| 3 | 60% | 100% | 40% | Posterior | 40 |
| 4 | 0% | 70% | 70% | Medial | 42 |
| 5* | 50% | 100% | 50% | Medial | 37 |
| 6* | 10% | 100% | 90% | Anterior | 40 |
| 7 | 0% | 80% | 80% | Posterior | 36 |

All slices were cut along the coronal plane. The amount of SCN in each slice was determined by the anteroposterior locations of the two coronal sections, i.e., from the anterior side (0%) to the posterior side (100%) of the SCN. MUA were recorded from the anterior, medial, or posterior part of the SCN. * indicates that neural activity of a subpopulation of the *in vitro* SCN neurons was available
